# Clinical application of metagenomic next-generation sequencing in rapid diagnosis and prognostic assessment of herpes simplex encephalitis

**DOI:** 10.3389/fmicb.2025.1534513

**Published:** 2025-03-25

**Authors:** Jin Tang, Ping Li, Haoming Xu, Jingzhe Han

**Affiliations:** ^1^Department of General Practice, The Second Affiliated Hospital of Xi’an Jiaotong University, Xi’an, Shaanxi, China; ^2^Department of Geriatric Respiratory, Hebei General Hospital, Shijiazhuang, Hebei, China; ^3^Department of Neurology, Hengshui People’s Hospital (Harison International Peace Hospital), Hengshui, Hebei, China

**Keywords:** Herpes Simplex Virus, encephalitis, cerebrospinal fluid, metagenomic next-generation sequencing, diagnose

## Abstract

**Purpose:**

Herpes simplex encephalitis (HSE) ranks among the most common causes of severe viral encephalitis. It leads to meningitis or encephalitis, with patients frequently encountering adverse outcomes. In this study, we utilized metagenomic next-generation sequencing (mNGS) to rapidly and accurately detect and identify the HSV pathogen directly from cerebrospinal fluid (CSF) samples, aiming to achieve a definitive diagnosis for encephalitis patients.

**Methods:**

From 2018 to 2023, we prospectively identified and enrolled 28 patients diagnosed with HSE at Hengshui People’s Hospital. CSF samples were subjected to mNGS to facilitate the diagnosis and characterization of HSE in this cohort. We compiled the clinical characteristics, supplementary examinations, and outcomes of HSE patients, with prognosis assessed using the Glasgow Outcome Scale (GOS) scores at discharge, 1 month post-discharge, and 3 months thereafter.

**Results:**

In this cohort of 28 patients, 12 were females and 16 males, with a mean age of 41.82 ± 18.23. HSE manifested with a variety of clinical symptoms, the most prevalent being headaches (67.9%), fever exceeding 38°C (60.7%), and altered consciousness (60.7%). Seizures (42.9%), vomiting (35.7%), and speech deficits (35.7%) were frequently observed, with a minority of patients displaying personality changes (28.6%). CSF analysis revealed pleocytosis and a mild increase in protein levels. Magnetic resonance imaging (MRI) abnormalities (28.6%) were primarily confined to the frontal and temporal lobes as well as limbic regions, with no indications of cerebral hemorrhage. Half of the patients exhibited Electroencephalogram (EEG) changes suggestive of encephalitis. HSE was confirmed through mNGS analysis of CSF within 3 days of admission. All patients received empirical treatment with ganciclovir, with 46.4% undergoing hormonotherapy and 32.1% receiving immunoglobulin therapy. At the three-month follow-up, 32.1% had GOS scores <5.

**Conclusion:**

HSE often presents with nonspecific signs of encephalitis, and it’s not easy for traditional CNS examinations to confirm the diagnosis. mNGS serves as a cutting-edge diagnostic tool for the rapid and precise identification of HSE, facilitating timely clinical diagnosis and intervention to prevent the progression of the disease.

## 1 Introduction

Herpes Simplex Virus (HSV), a ubiquitous double-stranded DNA virus, is taxonomically classified under the Alphaherpesvirinae subfamily within the Herpesviridae family. Characterized by its neurotrophic properties, HSV is primarily differentiated into two distinct serotypes: HSV-1 and HSV-2 based on their antigenic disparities (Aubert et al., 2024). Herpes simplex encephalitis (HSE) is an acute infectious disease of the central nervous system caused by the HSV, with 90% of cases attributed to HSV-1 (Zhang and Casanova, 2024). HSV-1 is a neurotropic pathogen that chiefly targets the oral cavity, pharynx, facial area, ocular tissues, and central nervous system (CNS). The global seroprevalence rate was approximately 60–95% in adults, making it the most prevalent infectious agent among individuals under the age of 60 (Rohani et al., 2023). HSE, a neurological complication, can lead to considerable CNS inflammation and neurologic dysfunction, manifesting a spectrum of clinical symptoms including cognitive deficits, aphasia, seizures, personality alterations, consciousness disturbances, and potentially culminating in coma (Ngan et al., 2024). It primarily impacts the temporal lobes, frontal lobes, and limbic system, resulting in hemorrhagic and necrotizing encephalitis (Armangué et al., 2023). Patients experiencing severe HSE may face complications stemming from extensive necrosis of the cerebral parenchyma, cerebral edema-related increases in intracranial pressure, and potentially lethal cerebrocranial herniation. Despite the administration of antiviral treatment, a significant number of HSE patients demonstrate enduring and debilitating neurological sequelae, highlighting the seriousness of the condition and the necessity for more effective and timely diagnosis and treatment (Sarton et al., 2021). Previous studies indicate that the fatality rate for patients with untreated HSE can soar up to 70%. Timely diagnosis and commencement of treatment prior to the onset of consciousness disturbances can significantly lower the mortality rate to between 17 and 30% (Gnann and Whitley, 2017).

Virus isolation and Polymerase Chain Reaction (PCR) analysis of Cerebrospinal Fluid (CSF) have emerged as the diagnostic gold standards for HSE. However, virus isolation is constrained by its lengthy procedure and low detection rate, while PCR is contingent upon the availability of nucleic acid sequences that can be effectively targeted by primers, thereby circumscribing the clinical utility of these methods (Meshref et al., 2024). Metagenomic Next-Generation Sequencing (mNGS) presents advantages such as independence from targeted primers, accelerated detection kinetics, and heightened diagnostic sensitivity, thereby facilitating the rapid and accurate etiological diagnosis of central nervous system infections (Benoit et al., 2024). Since the initial report of mNGS applications in 2014, this technique has been progressively integrated into the diagnostic arsenal for CSF pathogen detection (Segawa et al., 2014). However, as more advanced diagnostic technologies evolve, atypical manifestations of HSE are increasingly acknowledged.

Our aim is to enhance the diagnostic proficiency of clinicians in identifying atypical manifestations of HSE by performing a thorough analysis of the clinical profiles, supplementary diagnostic tests, and prognostic outcomes of HSE patients diagnosed via mNGS. This systematic evaluation emphasizes the pivotal role of mNGS in diagnostic stewardship, enabling the swift identification of HSE, the initiation of targeted therapeutic measures, and ultimately, the improvement of patient outcomes.

## 2 Materials and methods

### 2.1 Research subjects

This retrospective study was carried out at Harrison International Peace Hospital in Hebei, China, from 2018 to 2023, encompassing a total of 28 patients. The study cohort consisted of individuals with HSE who had one or more HSV sequences identified through mNGS of CSF. Exclusion criteria included: (1) Patients with concurrent central nervous system tumors; (2) Patients with encephalitis resulting from multiple pathogen infections; (3) Patients with autoimmune encephalitis; (4) Patients with incomplete clinical records. Follow-up assessments were subsequently arranged for the enrolled participants at 1 and 3 months following hospital discharge.

The research was approved and carried out under the supervision of the Harrison International Peace Hospital Ethics Committee, Hengshui, Hebei (approval number: 2023126).

### 2.2 Clinical data collection

We meticulously gathered comprehensive patient data, including demographic characteristics such as age and sex, as well as detailed medical histories with a particular focus on diabetes mellitus. Clinical manifestations and physical examination findings were systematically documented to provide a holistic view of the patient’s condition. We also carefully recorded the exact time of hospital admission and the interval between symptom onset and lumbar puncture to facilitate accurate temporal analysis. Laboratory investigations including complete blood count and CSF analysis, were conducted to obtain essential baseline data. We were actively engaged in neuroimaging studies using MRI, and EEG was conducted whenever clinically indicated. The results of CSF metagenomic testing were meticulously analyzed to guide our clinical decisions. Therapeutic interventions, including empirical antibiotic therapy, antiviral agents, corticosteroids, and immunoglobulin administration, were closely monitored to ensure optimal patient care. Clinical outcomes were rigorously evaluated, with a particular emphasis on length of hospital stay and mortality rate. In the follow-up assessments, post-discharge complications were carefully documented to provide a comprehensive view of the patient’s recovery journey.

Upon admission, we evaluated the patient’s state of consciousness using the Glasgow Coma Scale (GCS). A score of 15 indicates full consciousness, while a score of 3 signifies a total loss of arousal and awareness. The lower the score, the more severe the impairment of consciousness and the graver the prognosis. Scores ranging from 13 to 14 suggest mild consciousness impairment, 9 to 12 indicate moderate consciousness impairment, and below 8 signifies coma (Teasdale et al., 2014).

### 2.3 mNGS of cerebrospinal fluid

All patients underwent mNGS based on CSF collected via lumbar puncture. The CSF mNGS procedure unfolded as follows: (1) Specimen Collection: 1–2 mL of CSF was gathered, aliquoted, and preserved at −80°C for 30 min prior to mNGS analysis. (2) Sample Extraction and Quality Control: Genomic DNA was isolated using a micro-sample genomic DNA extraction kit (DP316, TIANGEN BIOTECH, Beijing, China) and fragmented to 200–300 base pairs (bp) utilizing a DNA cutting ultrasonic disruptor (Bioruptor Pico, Diagenode, Belgium). Fragment sizes were evaluated with a 2100 Bioanalyzer, and DNA library concentration was quantified by qPCR. (3) Library Construction: DNA libraries were assembled with end-repair, poly(A)-tailing, adapter ligation, and PCR amplification, followed by roller amplification by 2–3 fold to create DNA nanospheres. (4) Sequencing: DNA nanospheres were sequenced on the BGISEQ-50 platform at Beijing Golden Key Gene Technology Co., Ltd.

Following quality control, low-quality and short reads were eliminated. The remaining high-quality sequences were aligned against the BWA human genome database, and unmatched fragments were compared with a microbial database to identify pathogens, concentrating on the type and quantity of viral sequences. A positive result was characterized as detecting more than one viral copy in the CSF.

Our pathogen database is meticulously compiled from sources such as the Manual of Clinical Microbiology and NCBI databases,^1^ and incorporates one exemplary high-quality strain per species. It presently encompasses 12,895 bacterial genomes, 11,120 viral genomes, 1,582 fungal genomes, 312 parasitic genomes, 184 mycoplasma, and 177 mycobacterium genomes, enabling precise pathogen identification in CSF samples.

## 3 Treatment and clinical prognostic assessment

All patients underwent standardized antiviral therapy, with some receiving additional corticosteroids and intravenous immunoglobulin. Patients’ outcomes were evaluated utilizing the Glasgow Outcome Scale (GOS) at discharge, 1 month after discharge, and 3 months following discharge. A GOS score of 5 signifies a favorable prognosis, while a GOS score below 5 denotes an unfavorable prognosis.

The outcomes were classified according to the Glasgow Outcome Scale (GOS) (Bewersdorf et al., 2019) summarized as Table 1.

**Table 1:** Glasgow Outcome Scale (GOS): scores and descriptions.

GOS score	Outcomes category	Description
1	Death	–
2	Vegetative state	Unconscious with basic physiological functions preserved
3	Severe disability	Dependent on others for daily living
4	Moderate disability	Independent living with functional limitations
5	Good recovery	No disability or only minor disability

### 3.1 Statistical methods

Data analysis was performed utilizing SPSS 27.0. Normally distributed continuous variables were expressed as means ± standard deviations (Mean ± SD), while non-normally distributed continuous variables were reported as medians accompanied by interquartile ranges (IQRs). Categorical data were displayed as counts and percentages.

## 4 Results

### 4.1 The characteristics of the patients

The clinical attributes of these 28 patients (12 females and 16 males) with HSE are encapsulated in Table 2. The average age of this cohort of 28 individuals is 41.82 ± 18.23 years, comprising 2 diabetics, all of whom are immunocompetent. HSV encephalitis presents as unusual encephalitic lesions, with fever, headache, and disturbance of consciousness emerging as the primary clinical manifestations. Within the subgroup exhibiting disturbance of consciousness, 4 patients display mild impairment, 8 exhibit moderate impairment, and 5 are in a state of coma, including 2 with a total loss of arousal and awareness. Furthermore, convulsions, vomiting, and Speech impairment are commonly noted, with a minority of patients showing personality changes.

**Table 2:** Clinical and epidemiological characteristics of HSE (*N* = 28).

Characteristic	N (%)/(means ± standard)
Female sex	12 (42.9)
Age (years)	41.82 ± 18.23
Diabetes	2 (7.1)
Fever>38°C	17 (60.7)
Headache	19 (67.9)
Vomiting	10 (35.7)
Personality changes	8 (28.6)
Speech impairment	10 (35.7)
Convulsions	12 (42.9)
Disturbance of consciousness	17 (60.7)
GCS 15	11 (39.3)
GCS 13–14	4 (14.3)
GCS 9–12	8 (28.6)
GCS <8	5 (17.9)

### 4.2 Laboratory and diagnostic tests of HSE

We employed CSF mNGS to ascertain the DNA sequence of HSV. The lumbar puncture pressure values, laboratory assessments, MRI, EEG, and HSV metagenomic sequencing counts of the patients are summaried in Table 3. Hematological findings indicate a mild leukocytosis, implying an infectious origin. The median duration from symptom onset to lumbar puncture for these 28 patients is 38 h. The WBC count of CSF is moderately elevated, with a predominance of lymphocytes. CSF analysis reveals mildly increased protein levels, while glucose and chloride levels remain within the normal range, collectively suggestive of CNS infection. CSF culture produced methicillin-resistant coagulase-negative Staphylococcus and Enterococcus in only one instance. In this study, a total of 17 patients underwent EEG assessments. Specifically, three patients exhibited normal EEG patterns. Five patients demonstrated mild EEG abnormalities, characterized predominantly by low-amplitude slow waves localized to the frontal and bilateral temporal regions. Four patients exhibited moderate EEG abnormalities, with irregular high-amplitude spikes and spike-and-slow-wave discharges observed in the frontal, temporal, and occipital regions. Finally, five patients had severe EEG abnormalities, characterized by slow and disorganized brain wave frequencies, along with a large number of temporal spikes, spike-and-slow-wave discharges, or epileptiform discharges. The result of cranial MRI was performed on 22 patients demonstrated a spectrum of findings. Five patients exhibited normal MRI findings without significant abnormalities. One patient showed small, chronic ischemic foci in the white matter of the bilateral frontal lobes. Six patients demonstrated MRI evidence of inflammatory swelling involving the bilateral temporal lobes, thalami, hippocampi, insulae, and cingulate gyri, suggesting an active inflammatory process in these regions. In addition, 10 patients exhibited abnormal MRI signals within the frontal lobes, temporal lobes, and corpus callosum.

**Table 3:** Clinical laboratory tests and examination results.

Laboratory features	N (%)/(median, IQR^*^ or means ± standard)
WBC in blood (3.5–9.5 × 10^9^/L)	9.42 (8.03, 10.32)
Time from onset to lumbar puncture, hours	38 (14.25, 163.75)
CSF pressure (mmH2O)	160 (140, 217.5)
CSF WBC (×10^6^ cells/L)	44.5 (2.00, 193.75)
CSF protein (0.15–0.45 mg/dL)	0.51 (2.85, 7.00)
CSF glucose (2.5–4.5 mg/dL)	3.90 ± 1.05
CSF Chloride (120–130 mmol/L)	121.25 (116.58, 123.3)
Positive bacterial culture	1 (3.6)
Abnormal EEG	17 (60.7)
Abnormal MRI	17 (60.7)
Chronic ischemic	1 (4.2)
Inflammatory swelling	6 (25)
Abnormal MRI signals	10 (41.7)
HSV reads by mNGS<10	18 (64.3)
HSV reads by mNGS>10	10 (35.7)

* IQR, Interquartile range; WBC, White blood cells.

All 28 patients eventually underwent mNGS through CSF, and the HSV nucleic acid sequences were accurately detected. Eighteen patients presented HSV sequence counts of less than 10. mNGS identified several pathogens of indeterminate clinical relevance. In addition to HSV, we identified *Moraxella osloensis*, *Staphylococcus*, *Corynebacterium*, *Micrococcus luteus*, and *Candida parapsilosis*. The reads counts and relative abundance of these pathogens were too low, and they are all part of the normal human microbiota, acting as opportunistic pathogens. In this study, all patients had immunocompetent. Laboratory tests and cerebrospinal fluid examinations indicated viral infections. Considering the patients’ encephalitis symptoms, these low-abundance microorganisms are more likely to be background or contaminating flora.

Furthermore, a few patients exhibited concurrent infections with Human Herpesvirus 6 (HHV-6), Epstein–Barr virus (EBV), and Cytomegalovirus (CMV). However, the reads counts and relative abundance of these viruses were significantly lower than those of HSV, with low confidence levels. In conjunction with the patients’ encephalitis symptoms, disease course, cranial MRI, EEG, and CSF results, HSV is considered the primary pathogen. Nevertheless, an examination of the clinical data for these individuals suggests that these viral findings are unlikely explanations for their clinical syndromes.

### 4.3 Treatment and prognosis of HSE

The treatment and prognosis details of the patients are encapsulated in Table 4. All our patients commenced antiviral therapy with ganciclovir approximately 5.08 days following the onset of illness. Among them, two patients received intravenous immunoglobulin for immunomodulatory therapy, six patients were treated with methylprednisolone or dexamethasone for anti-inflammatory purposes, and seven patients underwent a combined regimen of corticosteroids and intravenous immunoglobulin.

**Table 4:** Treatment and prognosis.

Treatment and prognosis	N (%)/ (median, IQR)*
Length of hospital stay	14 (11, 21.5)
Time from onset to medication, days	5.08 (3.52, 11.06)
Empiric treatment with ganciclovir	28 (100)
Hormonotherapy	13 (46.4)
Immunoglobulin	9 (32.1)
GOS <5 at discharge	18 (64.3)
GOS <5 at 1 month post-discharge	11 (39.3)
GOS <5 at 3 month post-discharge	7 (32.1)

*IQR, Interquartile range.

Our patients were followed up for a total of 3 months at outpatient. The number of patients experiencing adverse outcomes gradually decrease at the time of discharge, 1 month post-discharge, and 3 months post-discharge. Upon discharge, the functional status of the patients varied significantly. One patient was in a state of unconsciousness, with only basic physiological functions preserved. Seven patients were unable to perform daily activities independently and required assistance from others. Ten patients were capable of living independently but had certain functional limitations, while another ten patients exhibited no significant disability or only minor impairments.

One month after discharge, there was notable improvement among the patients. One patient remained unconscious with basic physiological functions intact. Two patients continued to rely on others for daily living. Eight patients were living independently despite having functional limitations, and 17 patients had either no disability or only minor impairments.

By 3 months after discharge, further progress was observed. One patient was still unconscious, and one patient remained dependent on others for daily activities. Three patients experienced limb weakness, and two patients had speech disturbances and cognitive decline. However, a significant majority of 21 patients had fully recovered to a normal state. Moreover, during our telephone follow-up this year, the one patient who was unconscious showed no significant improvement. One patient with severe disability had improved to moderate disability and was able to live independently with some functional limitations. Three patients with moderate disability experienced improvement in limb weakness and speech disturbances.

## 5 Discussion

HSE, primarily induced by HSV-1, can occur at any age and is generally sporadic, displaying no specific seasonal inclination. It is acknowledged as the most prevalent cause of encephalitis among viral origins, with an annual incidence estimated to range from 1 in 250,000 to 1 in 500,000 (Hussain et al., 2024). HSV is acknowledged as the primary pathogen in adult viral encephalitis, with cases of encephalitis exceeding those of meningitis. Prior to the encephalitic episode, patients often exhibit nonspecific prodromal symptoms, including respiratory infections, fever, headache, and fatigue. Thereafter, within days of onset, patients may experience more distinct neurological manifestations such as insomnia, alterations in behavior or personality, seizures, or coma (Bradshaw and Venkatesan, 2016). Patients may initially exhibit neuropsychiatric manifestations and alterations in personality, accompanied by a range of neurological impairments (Narasimhappa et al., 2024). In our case series of 28 patients, the primary manifestations were nonspecific, encompassing fever, headache, and vomiting. Notably, none of these patients had a history of diabetes mellitus, and all were under the age of 60, indicating that the lack of a prior history of diabetes and a younger age at admission should not exclude the consideration of HSE. Within our patient cohort, convulsions and personality alterations as the initial symptoms were frequently accompanied by speech impairments and disturbances in consciousness, predominantly presenting as moderate consciousness disruption. Brain MRI consistently revealed frontotemporal edema or inflammatory changes in 6 cases, which strongly indicate the involvement of the frontotemporal lobes. HSV primarily impacts the frontotemporal lobes and limbic system, resulting in brain injury, cerebral edema, and potentially hemorrhagic and necrotic brain lesions. Before the emergence of antiviral treatment, HSE was marked by a significant incidence and mortality rate (Ngan et al., 2024). Patients exhibiting these symptoms are at considerable risk of advancing to severe illness, emphasizing the necessity for increased clinical alertness among physicians. Clinicians should also be more assertive in commencing antiviral treatment as promptly as possible. Nevertheless, it is important to note that there were five patients whose brain MRI scans showed no significant abnormalities, and another 11 patients exhibited atypical changes on brain MRI. These findings collectively underscore the fact that non-specific changes on brain MRI cannot definitively rule out the possibility of HSE.

In patients with HSE, CSF analysis reveals a pleocytosis with nucleated cell counts ranging from (50 to 100) × 10^6/L, with the upper limit potentially reaching 1,000 × 10^6/L. Protein levels are noted to be mildly to moderately elevated, while glucose and chloride levels remain within the normal range (Ralph et al., 2024). Our study findings correspond with the existing literature, demonstrating a moderate increase in CSF WBC count with a predominance of lymphocytes, mild elevation of protein, and normal concentrations of glucose and chloride, strongly suggesting the likelihood of HSE.

EEG is an exceptionally sensitive and objective technique capable of precisely forecasting brain injury and its severity, mirroring the functionality of neural cells. It serves as a crucial diagnostic instrument for HSE and aids in improving the precision of patient evaluation (Hersh et al., 2023). In this study, 60.7% of the patients displayed abnormal EEGs, primarily characterized by widespread high-amplitude slow waves in the frontal and temporal lobes, accompanied by sharp waves and spike waves or epileptiform discharges. These observations align with the frontal and temporal lobe lesions identified in brain MRI, both indicating the presence of encephalitis. This corroborates the findings reported by Jeantin et al. (2023). However, it is also observed that three of the patients exhibited normal EEG results, suggesting that a normal EEG cannot dismiss the possibility of HSE.

While clinical manifestations, imaging examinations, CSF analysis, and EEG constitute the foundation for diagnosing HSE, the conclusive diagnosis of HSE ultimately depends on the affirmative results of HSV nucleic acid identification. The conventional detection of HSV positivity in cerebrospinal fluid through PCR remains the gold standard for HSE (Devireddy et al., 2022). Conversely, mNGS technology can detect pathogenic microorganisms in brain tissue and CSF objectively, remaining impervious to clinical interventions, especially antibiotics, and substantially reduces the diagnostic timeframe to within 3 days (Zhang et al., 2024), particularly for patients whose typical encephalitis assessments, encompassing clinical manifestations, imaging evaluations, cerebrospinal fluid analyses, and electroencephalography, are atypical. In contrast to PCR, which presents limitations such as the necessity for specific primer formulation, susceptibility to contamination during enrichment processes, and a constrained detection range that fails to recognize novel pathogens, mNGS serves as a vital alternative for clinicians to promptly diagnose HSE. We noted that all patients underwent their initial lumbar puncture and mNGS testing roughly 3 days following hospital admission. Among them, 18 individuals exhibited a read count of less than ten. These patients had a relatively younger mean age, and their CSF white blood cell count, glucose, and protein levels were significantly lower. Additionally, the prevalence of unfavorable outcomes at discharge, 1 month post-discharge, and 3 months post-discharge was relatively minimal. However, there was no statistically significant difference between the groups. This may suggest that viral load is positively correlated with the inflammatory response, clinical manifestations, and prognosis of the disease. However, additional validation with a larger sample size is necessary to substantiate these findings. This swift turnaround time highlights the crucial role of mNGS in detecting pathogenic CNS infections, thereby emphasizing the importance of the technology. Although we did not perform PCR validation, existing literature suggests that CSF HSV-specific PCR may not improve the sensitivity for diagnosing HSV CNS infections when mNGS is conducted on CSF samples. CSF mNGS is the most sensitive microbial assay for diagnosing HSV central nervous system infections and can unexpectedly uncover pathogens that traditional diagnostic tests may overlook (Zhou et al., 2022). mNGS facilitates pathogen detection in an unbiased manner, independent of prior knowledge regarding the target microorganism. Given optimal sequencing read lengths and multiple concordant alignments to the microbial genome, mNGS is theoretically capable of precisely identifying the etiologic agent of infection (Wang et al., 2020). Despite the fact that mNGS is a costly examination requiring specialized apparatus and laboratory facilities, which may impedes the extensive adoption of this technology, the expenses associated with this testing are decreasing annually due to continuous technological progress. It is anticipated that in the foreseeable future, the costs will align with a more acceptable range, facilitating its wider application in clinical settings.

Ultimately, all our patients were administered ganciclovir following the onset of illness, with some individuals receiving concurrent steroid and/or immunoglobulin therapy, leading to a favorable prognosis in nearly 79.9% of the patients. In the management of HSV infections, the utilization of antiviral medications such as ganciclovir and cidofovir has been shown to effectively control viral replication and diminish the severity of the disease progression. Our study also confirmed that the overall therapeutic effect and prognosis are favorable. The combination of corticosteroids and immunoglobulin therapy may assist in alleviating inflammatory responses and bolstering the body’s immune response, thereby further enhancing therapeutic outcomes. Despite these advancements, additional clinical trials are necessary to evaluate the long-term efficacy and safety of these treatment protocols (Kawamura et al., 2024).

Finally, our study has certain limitations. Future research will expand the sample size and utilize PCR validation with group comparisons to explore the correlation between mNGS read counts and the prognosis of HSE. Additionally, we will conduct a more in-depth analysis of the factors influencing prognosis, evaluate treatment outcomes over a longer follow-up period, and perform a comprehensive analysis of disease characteristics. These efforts aim to enhance early diagnosis and treatment, thereby improving patient outcomes.

## 6 Conclusion

HSE is frequently characterized by non-specific encephalitic symptoms, posing significant challenges to early diagnosis. Traditional diagnostic modalities, including cranial MRI, EEG, and routine CSF analysis, can be inconclusive. mNGS has transformed HSE diagnosis by rapidly and accurately detecting HSV through CSF. It enables early targeted antiviral and immunomodulatory treatments, preventing disease progression and improving outcomes. Thus, mNGS is a vital tool in modern HSE management.

## Data Availability

The original contributions presented in the study are publicly available. This data can be found here: http://www.ncbi.nlm.nih.gov/bioproject/1233591.

## Footnotes

^1^ ftp://ftp.ncbi.nlm.nih.gov/genomes/
